# Transcriptomic and metabolomic characterization of post-hatch metabolic reprogramming during hepatic development in the chicken

**DOI:** 10.1186/s12864-021-07724-w

**Published:** 2021-05-24

**Authors:** Heidi A. Van Every, Carl J. Schmidt

**Affiliations:** 1 Center for Bioinformatics and Computational Biology, University of Delaware, Newark, Delaware, USA; 2 Department of Animal and Food Sciences, University of Delaware, Newark, Delaware, USA

**Keywords:** High-throughput, Cell proliferation, Metabolic reprogramming, Organ growth, Pathway, Hypoxia, Glycolysis, Lipogenesis, Regulation

## Abstract

**Background:**

Artificial selection of modern meat-producing chickens (broilers) for production characteristics has led to dramatic changes in phenotype, yet the impact of this selection on metabolic and molecular mechanisms is poorly understood. The first 3 weeks post-hatch represent a critical period of adjustment, during which the yolk lipid is depleted and the bird transitions to reliance on a carbohydrate-rich diet. As the liver is the major organ involved in macronutrient metabolism and nutrient allocatytion, a combined transcriptomics and metabolomics approach has been used to evaluate hepatic metabolic reprogramming between Day 4 (D4) and Day 20 (D20) post-hatch.

**Results:**

Many transcripts and metabolites involved in metabolic pathways differed in their abundance between D4 and D20, representing different stages of metabolism that are enhanced or diminished. For example, at D20 the first stage of glycolysis that utilizes ATP to store or release glucose is enhanced, while at D4, the ATP-generating phase is enhanced to provide energy for rapid cellular proliferation at this time point. This work has also identified several metabolites, including citrate, phosphoenolpyruvate, and glycerol, that appear to play pivotal roles in this reprogramming.

**Conclusions:**

At Day 4, metabolic flexibility allows for efficiency to meet the demands of rapid liver growth under oxygen-limiting conditions. At Day 20, the liver’s metabolism has shifted to process a carbohydrate-rich diet that supports the rapid overall growth of the modern broiler. Characterizing these metabolic changes associated with normal post-hatch hepatic development has generated testable hypotheses about the involvement of specific genes and metabolites, clarified the importance of hypoxia to rapid organ growth, and contributed to our understanding of the molecular changes affected by decades of artificial selection.

**Supplementary Information:**

The online version contains supplementary material available at 10.1186/s12864-021-07724-w.

## Background

The modern broiler (meat) chicken is the product of more than 60 years of artificial selection for commercially desirable traits, resulting in both improved feed efficiency and breast muscle yield. Currently, broilers reach market weight in ¾ the time it took in the 1950s, yet they weigh nearly twice as much as the 1950s breeds, with the breast muscle representing a greater component of the overall bird mass [1]. Several studies have compared modern lines with unselected lines in terms of growth rate and feed efficiency [2, 3]. In one such study comparing growth of a modern broiler line (Ross 708) with a legacy line of commercial general-purpose birds unselected since the 1950s (UIUC) over the first 5 weeks post hatch, the breast muscle was found to comprise 18 and 9% of total body mass, respectively [4]. Additional changes in growth pattern manifest in liver allometry. In both lines, the relative liver mass reached a similar maximum of approximately 3.8% of body mass and then began declining. However, this peak occurred a week earlier in the modern broiler. This finding provided part of the basis for this study, including selection of the liver and first 3 weeks post hatch, as it was hypothesized the earlier onset of this peak arose due to selection for rapid growth and the liver’s important role in nutrient metabolism.

Chicks undergo drastic physiological changes as a consequence of hatching. The developing embryo relies entirely on nutrients from the yolk [5–7]. During late embryonic development, much of the yolk lipid is absorbed and stored in the liver, predominately as cholesteryl esters [8]. At day 18 of incubation, 3 days prior to hatch, lipids make up 10% of the liver’s mass due to absorption and storage of yolk nutrients [9]. This stored lipid, along with the yolk remnant, provides the chick with a nutrients following hatch, but by day 5 post-hatch 90% of the yolk lipid has been absorbed [10]. Chicks are provided with a carbohydrate-rich diet at hatch because fasting during this period stunts the early muscle growth potential of chicks [11]. These early changes in nutrient source, coupled with rapid growth, mean maintaining metabolic homeorhesis is a major challenge facing the liver in the early weeks following hatch.

High-throughput transcriptome analyses provide snapshots of transcribed RNAs at any given time and are useful to identify differentially regulated genes between conditions or time points. Combining transcriptomics with untargeted metabolomics is a powerful means to infer hypotheses about the interactions between the transcriptome and metabolome. For example, integrating these two high throughput methods identified metabolic and signaling pathways responding to heat stress in the liver of modern broilers [12]. Previous studies have described the hepatic transcriptome of the modern broiler [13–16]. One study compared the hepatic transcriptome over six time points during the embryo to hatchling transition, from 16-day embryos to 9-day old chicks [17]. They identified many metabolic pathways consistent with the nutrient source transition the chicks undergo in the first week post hatch, especially some affecting lipid metabolism. Another recent study examined changes in the hepatic transcriptome resulting from immediate post-hatch fasting and re-feeding, identifying genes regulated by lipogenic transcription factor THRSPA and switching between lipolytic and lipogenic states [18].

There have been no integrated high-throughput studies of the modern broiler liver under normal conditions in the critical first 3 weeks post-hatch. Thus, the molecular changes that are occurring during this time period – the metabolic drivers of rapid muscle growth and feed efficiency – are poorly understood. Exploring these in a data-driven fashion can elucidate new knowledge about the liver’s functions during early post-hatch growth of the chick, and also how the liver itself is developing. In this work, by integrating the hepatic transcriptome and metabolome, we compare the core metabolic pathways of the liver at two time points: Day 4 (D4) and Day 20 (D20) post-hatch. These were selected to capture the metabolic reprogramming required to support the transition from relying on stored yolk to orally consumed feed that underlies the growth rate and phenotype of the modern broiler.

## Results

### Phenotypic measurements and i-STAT blood chemistry

**Fig. 1:**
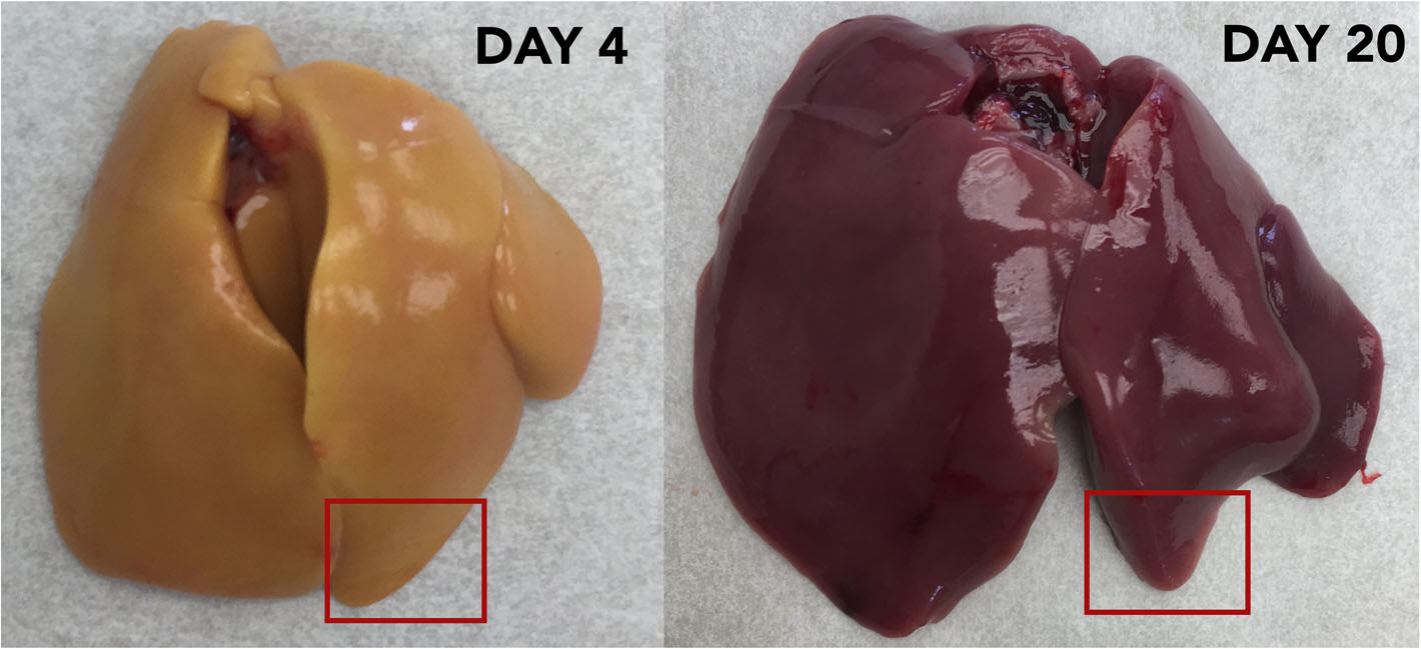
Contrast in liver color at D4 and D20 post-hatch. The yellow color at hatch is indicative of the absorption and storage of yolk lipid and nutrients that occurs during late embryonic development. The liver gradually changes to deep red as the chick grows, concurrent with the depletion of the liver’s stores. Tissue was routinely sampled from the lower left lobe, as indicated by the red boxes. Note: Liver sizes are not on the same scale

**Fig. 2:**
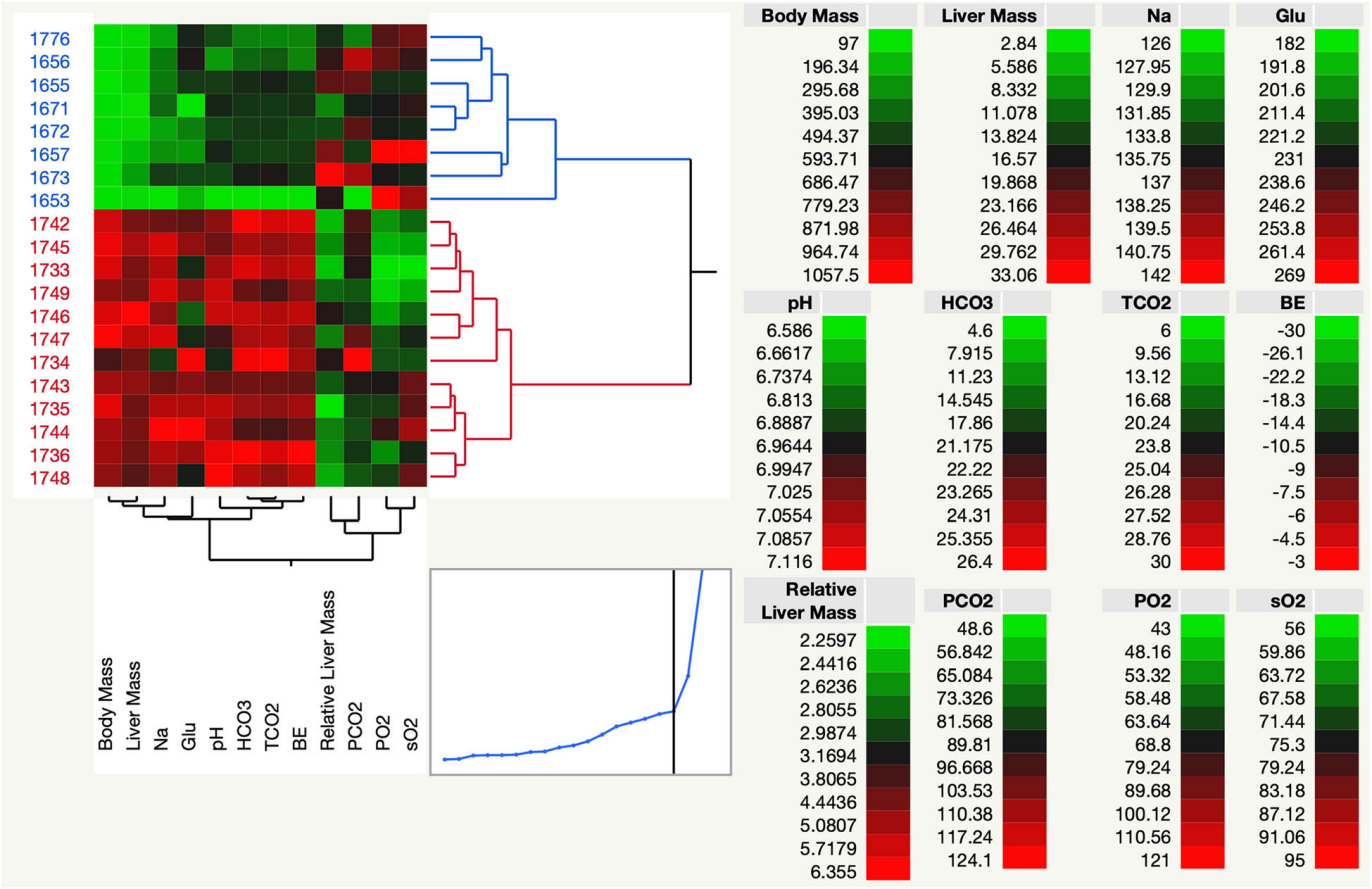
Hierarchical clustering of morphometric and blood chemistry measurements from all birds. There were no i-STAT readings from three D4 birds, and all D20 birds are included regardless of quality elimination from transcriptome analysis

At D4 post-hatch, the liver was noticeably yellow in color, gradually changing to deep red by D20 (Fig. 1). Mean phenotypic measurements of bird growth, liver allometry, and i-STAT blood chemistry values are shown in Table 1; Fig. 2 shows hierarchical clustering of this data, which separates the two groups by age. Body mass and liver mass showed the largest difference between days and were positively correlated with bird age (PCC 0.98 and 0.97, respectively). Relative liver mass was negatively correlated with bird age (PCC − 0.51). The top blood chemistry values positively correlated with bird age were sodium (Na, PCC 0.89), bicarbonate (HCO_3_, PCC 0.79), total carbon dioxide (TCO_2_, PCC 0.77), and pH (PCC 0.75). Partial oxygen (PO_2_, PCC − 0.70) and oxygen saturation (sO_2_, − 0.56) were negatively correlated with bird age.

**Table 1:** Summary of phenotypic trait and blood gas values by day, along with published references for comparison

	Median		Mean + _ Standard Deviation		***p*** value	Variable trend with age	Adult Breeder Values [19]	
	D4	D20	D4	D20			Range	Mean
**Body Mass (g)**	112.25	987.50	110.75 ± 5.54	912.64 ± 134.37	< 0.0001	+	NA	NA
**Liver Mass (g)**	3.77	23.35	4.29 ± 1.43	25.01 ± 4.28	< 0.0001	+	NA	NA
**Normalized Liver Mass (%)**	0.034	0.027	0.039 ± 0.012	0.028 ± 0.004	0.0214	–	NA	NA
**pH**	6.88	7.08	6.83 ± 0.13	7.05 ± 0.06	0.0034	+	7.28–7.57	7.42
**PCO**_**2**_ **(mm Hg)**	87.70	84.40	87.47 ± 23.51	91.03 ± 16.91	0.7513	NA	25.9–49.5	37.7
**PO**_**2**_ **(mm Hg)**^**a**^	82.00	61.00	88.29 ± 23.45	55.71 ± 9.16	0.0021	–	32.0–60.5	46.2
**HCO**_**3**_ **(mmol/L)**	17.90	24.80	15.39 ± 5.49	24.77 ± 1.05	0.0037	+	18.9–30.3	24.6
**Base Excess (BE)**^**a**^	−14.50	−6.00	−16.17 ± 3.82	− 5.86 ± 0.9	0.0031	+	−6.8 - 7.2	0.2
**sO**_**2**_ **(%)**	78.00	70.00	81.14 ± 7.73	70.29 ± 9.67	0.0398	–	70.6–93.3	82
**Glu (mg/dL)**	206.00	230.00	208.57 ± 18.79	238.86 ± 19.04	0.0112	+	207.2–260.7	234
**TCO**_**2**_	20.00	28.00	17.71 ± 6.02	27.57 ± 1.51	0.0044	+	19.9–31.5	25.7
**Na (mmol/L)**^**a**^	130.00	140.00	130.14 ± 2.54	139.14 ± 2.41	0.0025	+	141.6–152.6	147.1

^a^ Denotes Wilcoxon test was used instead of t-test

TCO_2_, PCO_2_, HCO_3_, and pH are used to assess blood acid-base balance, which is maintained by the kidneys and lungs and affected by both metabolism and respiration. TCO_2_ is a measure of total blood carbon dioxide while PCO_2_ measures the difference between CO_2_ produced by the cells and removed through respiration. HCO_3_ is a blood buffer produced by the kidneys, representing the metabolic component of acid-base balance. Given a change in blood pH due to any of these values, BE can help to differentiate between respiratory or metabolic causes. It is calculated as the difference between titratable base and titratable acid, and not susceptible to respiratory factors such as changes in PCO_2_. An increase in pH was observed from D4 to D20, indicating a shift in acid-base balance as the birds age. The metabolic measures of acid-base balance (buffer HCO_3_ and BE) were increased from D4 to D20, while the respiratory component was unchanged (PCO_2_), indicating the shift in acid-base balance is largely due to metabolic factors.

### Transcriptome analysis: top 100 abundant transcripts from each day

Examination of the 100 most abundant transcripts expressed in either the D4 or D20 liver (total of 200) identify important similarities in functions at these two time points. Of these genes, 88 were common between both D4 and D20. Enriched Gene Ontology (GO) terms among these common genes included Translation, encompassing 14 ribosomal proteins and Secretory Vesicle, which included albumin along with proteins involved in lipid transport, complement and coagulation. Two other enriched GO terms shared by both days were Mitochondria and Oxidative Phosphorylation. These terms were enriched by genes encoding mitochondrial rRNAs and tRNAs along with NADH dehydrogenases, cytochrome oxidases and ATP synthase subunits. One gene product unique to D20 encodes glucose 6-phosphatase (G6PC) an enzyme critical to gluconeogenesis. Several transcripts encoding genes affecting additional processes were found in the D4 top 100 list that were not in that D20 list (Tables S1A & S1B). These include proteins involved in lipid metabolism and transport, amino acid catabolism, peptidase inhibitors, a sulfotransferase and hemoglobin A. These results indicate that, despite the changes undergone by the liver from D4 to D20 the major hepatic functions such as production of complement proteins, or secretion of albumin, are preserved between time points.

### Transcriptome ontology analysis by day

**Fig. 3:**
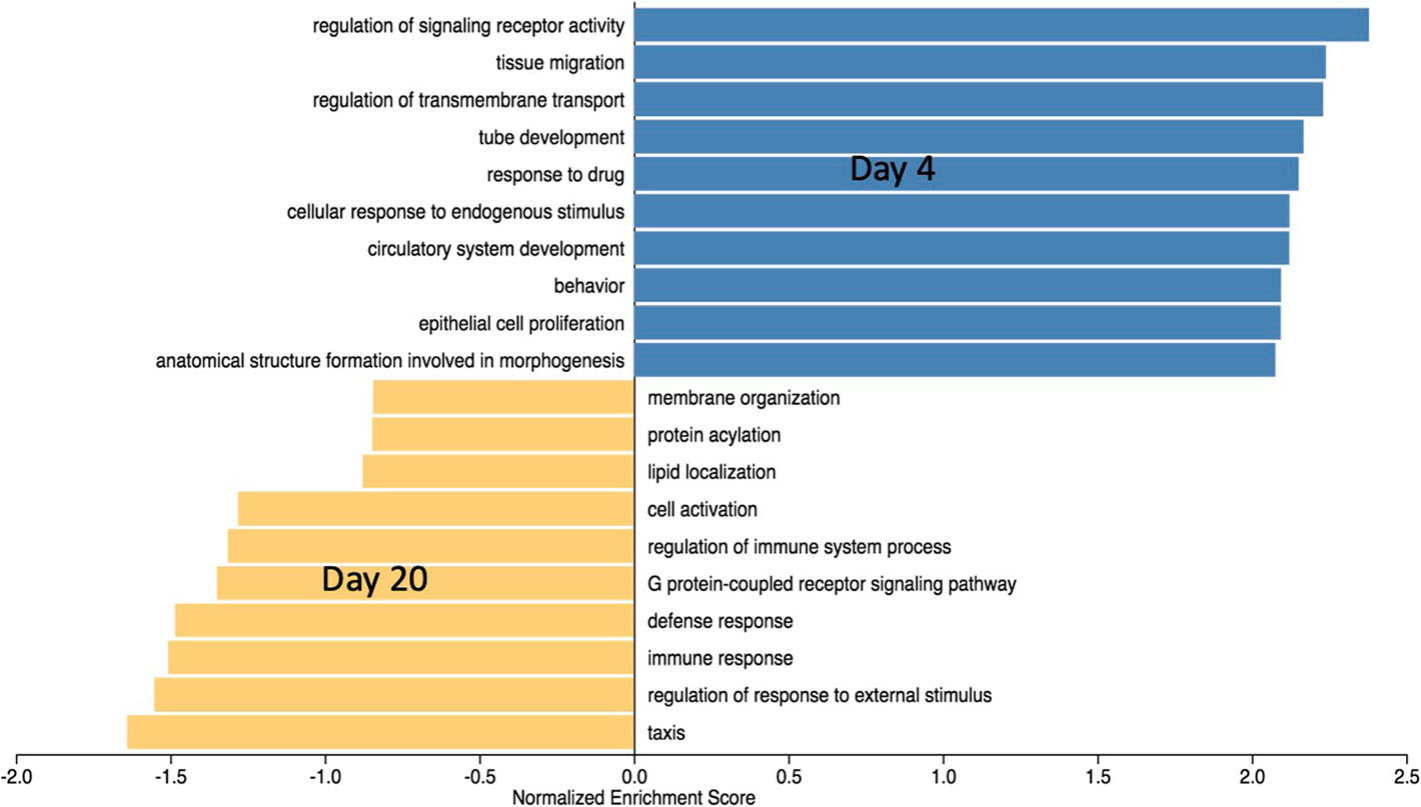
Gene Ontology Biological Process Terms enriched at either Day 4 (blue) or Day 20 (gold)

Ontology enrichment analysis using DAVID [20, 21] showed distinct differences between time points (Fig. 3). At D4, top Functional Annotation Clusters were related to a variety of cell cycle elements including mitosis, cell division, centromeric chromosome condensation & segregation, DNA replication, and transitions between cell cycle phases. Other clusters contained terms involved in ribonucleotide binding, kinase activity, amino-acid modification, vasculature development, and migration and motility of epithelial cells. At D4, the top enriched KEGG pathway from STRING [22, 23] was “Cell Cycle,” with 36 out of 123 proteins represented. DNA replication and cellular senescence were also among the top ten. Purine and Pyrimidine metabolism was the only metabolic pathway enriched by the transcriptome at D4. At D20, top Functional Annotation Clusters were related to immune response, including T cell and B cell receptor signaling pathways, toll-like receptor signaling pathway, immune cell aggregation, activation, proliferation, and differentiation. One cluster contained terms related to oxidoreductase activity including heme binding and cytochrome P450. The top enriched KEGG pathway at D20 was “Metabolic Pathways,” with 162 out of 1250 proteins represented. Other enriched pathways were related to carbohydrate metabolism, including fructose and mannose, and galactose, and immune-related pathway Th17 cell differentiation. Ontology and pathway analysis of the transcriptome gave the first glimpse of the major processes important to the liver at each time point: rapid organ growth and vasculature development at D4; carbohydrate metabolism and immune cell population expansion at D20.

### Hypoxic environment at D4

**Fig. 4:**
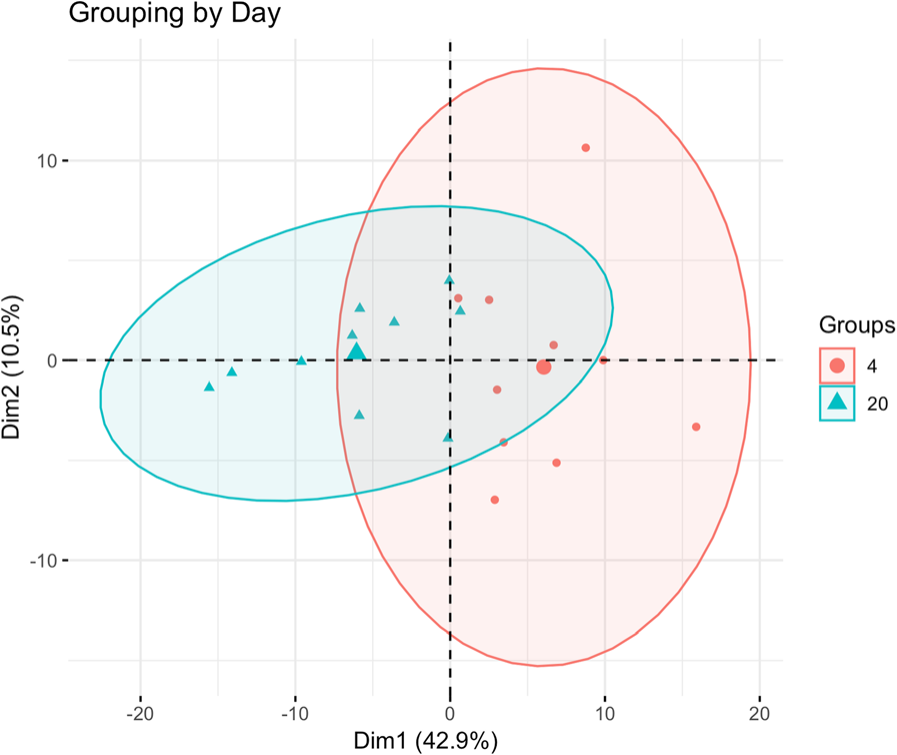
PCA of hypoxia genes showing clear separation by day along Dimension 1

Early in the process of investigating the data, it was noticed that HIF1A transcripts were elevated in the D4 liver (log_2_ fold change 0.56, adjusted *p*-value 0.03), suggesting the tissue is under hypoxic conditions. To further evaluate this possibility, a list of human genes induced under hypoxic conditions was downloaded from the Gene Set Enrichment Analysis resource [24, 25] and used to extract the orthologs from the D4 and D20 expression data. Principal component analysis revealed that 43% of the variance was associated with the day post-hatch; with the D4 samples showing elevated levels of many of the transcripts associated with hypoxia (Fig. 4, Table S2).

### Metabolome analysis: PCA, random forest, and top significant metabolites

**Fig. 5:**
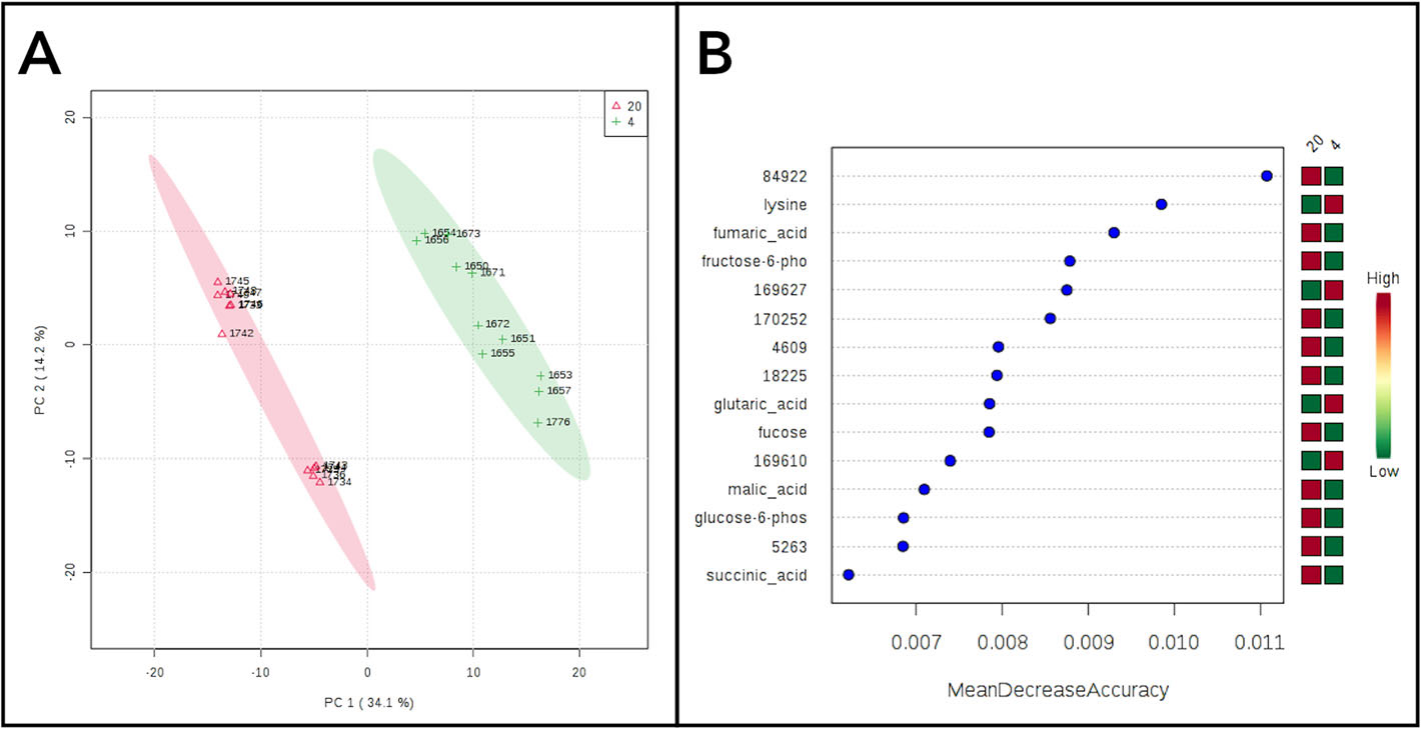
**a** PCA showing clear separation of individuals by top metabolites. D4 = green, D20 = red. **b** Top metabolites contributing to random forest classification that correctly separated D4 and D20. Compound 84,922 was identified by PubChem ID as cytidylic acid (CMP)

Principal component analysis of metabolites separated D4 birds from D20 birds (Fig. 5a), and random forest also correctly classified birds by age group. The top compounds contributing to random forest classification are shown in Fig. 5b. The top identified compounds contributing to random forest classification included two more abundant at D4 (lysine, glutaric acid) and seven more abundant at D20 (CMP, fumaric acid, fructose-6-phosphate, fucose, malic acid, glucose-6-phosphate, succinic acid). Lysine is an essential amino acid important for growth, and glutaric acid is a byproduct of amino acid metabolism. Fumaric acid, malic acid, and succinic acid are TCA cycle intermediates, while fructose-6-phosphate, glucose-6-phosphate, and fucose are sugars involved in glycolysis and other carbohydrate metabolic pathways. CMP (Cytidine monophosphate), is a pyrimidine-derived nucleotide.

By t-test, 90 compounds were more abundant at D4 and 112 at D20. Some of the top most significant compounds by log_2_ fold change and *p*-value are detailed in Table 2. At D4, several of the top significant metabolites were yolk-derived nutrients and fatty acids including retinal, oleic acid, palmitoleic acid, and gamma-tocopherol (Vitamin E). Retinal, a retinoid derived from known egg yolk nutrient Vitamin A, is critical in numerous processes including growth regulation and lipid metabolism [26]. The second most significant compound, 2-hydroxybutanoic acid, can be produced as a byproduct of threonine catabolism and glutathione synthesis, and is also part of propanoate metabolism [27]. Lactobiose (lactose), while most commonly known as a milk sugar, is a common chicken feed additive. It is a disaccharide comprised of glucose and galactose, and can serve as a source of glucose. Phosphoserine is an intermediate of amino acid metabolism, and uric acid is the major waste product of protein catabolism in birds. Phosphoenolpyruvate and 3-phosphoglycerate are intermediates of glycolysis that are also involved in several other metabolic pathways including the TCA cycle and lipid metabolism. Phosphoenolpyruvate can be generated from TCA cycle intermediate oxaloacetate and may reflect utilization of alternative carbon sources. Uracil is an RNA pyrimidine nucleobase. In the liver, as UDP-glucose, it has roles in carbohydrate metabolism where it regulates the conversion of glucose to galactose [28].

**Table 2:** Top significant identified metabolites with pathway membership or role in metabolism. Lipid and amino acid metabolism-related compounds predominated in D4, while many of those present in D20 were involved in carbohydrate metabolism

Compound	Fold-change (Log2)	Adjusted ***p***-value	Day	Pathway
Retinal	4.42	2.22E-10	D4	Vitamin A
2-Hydroxybutanoic Acid	3.34	4.25E-03	D4	Amino Acid-Glutathione Metabolism
Oleic acid	3.12	2.41E-3	D4	Lipid metabolism
Palmitoleic acid	2.87	5.98E-9	D4	Lipid metabolism
Lactobiose (lactose)	2.60	3.11E-5	D4	Carbohydrate metabolism
Phosphoserine	1.99	6.92E-5	D4	Serine metabolism
Uric Acid	1.79	1.03E-6	D4	Nitrogen metabolism
Phosphoenolpyruvate	1.77	1.11E-4	D4	Glycolysis (ATP synthesis phase)
Gamma-Tocopherol	1.73	2.73E-4	D4	Vitamin E metabolism
Uracil	1.66	1.59E-6	D4	Pyrimidine metabolism
3-Phosphoglycerate	1.63	3.67E-3	D4	Glycolysis (ATP synthesis phase)
Aspartate	−1.57	1.6E-4	D20	Amino acid metabolism
Adenosine	−1.64	5.7E-3	D20	Purine metabolism
Guanosine	−1.65	4.3E-3	D20	Purine metabolism
Hypoxanthine	−1.74	3.13E-4	D20	Purine metabolism
Creatinine	−1.82	7.65E-3	D20	Creatine metabolism
Citrate	−2.00	3.41E-5	D20	TCA cycle
Fructose-6-Phosphate	−2.15	4.86E-7	D20	Gluconeogenesis or Glycolysis (ATP incorporating phase)
CMP	−2.42	2.53E-7	D20	Pyrimidine metabolism, TAG, lipid & sialic acid synthesis
Inosine	−2.66	1.21E-5	D20	Nucleoside metabolism
5-Methoxytryptamine	−2.81	9.43E-7	D20	Tryptophan metabolism
Hexose-6-Phosphate	−3.25	5.51E-8	D20	Carbohydrate metabolism
Succinate	−3.35	2.64E-9	D20	TCA cycle
Glucose-6-Phosphate	−3.48	1.44E-6	D20	Gluconeogenesis or Glycolysis (ATP incorporating phase)
Fumarate	−4.87	1.9E-9	D20	TCA cycle
Malate	−5.32	9.28E-12	D20	TCA cycle

In D20, several of the most significant identified metabolites were intermediates of the TCA cycle (malic acid, fumaric acid, succinic acid, citric acid), or sugars involved in carbohydrate metabolism (glucose-6-phosphate, hexose-6-phosphate, fructose-6-phosphate). Adenosine, guanosine, and inosine are nucleosides. CMP and hypoxanthine are also part of purine and pyrimidine metabolism. 5-methoxytryptamine is derived from serotonin, a neurotransmitter derived from tryptophan. Creatinine is a waste product of amino acid catabolism in muscle. Aspartate is a non-essential amino acid.

Metabolome results show enrichment in lipids, vitamin A, vitamin E, carbohydrate, serine, cysteine, uric acid and uracil metabolism as metabolic characteristics of D4 post-hatch liver. In contrast, D20 metabolome data show enrichment of the TCA cycle, gluconeogenesis (or glycolysis) pathways along with aspartate, tryptophan, creatine, purine, pyrimidine, and inosine metabolism.

### Metabolic pathway-level integration of transcriptome and metabolome

#### Carbohydrate metabolism

**Fig. 6:**
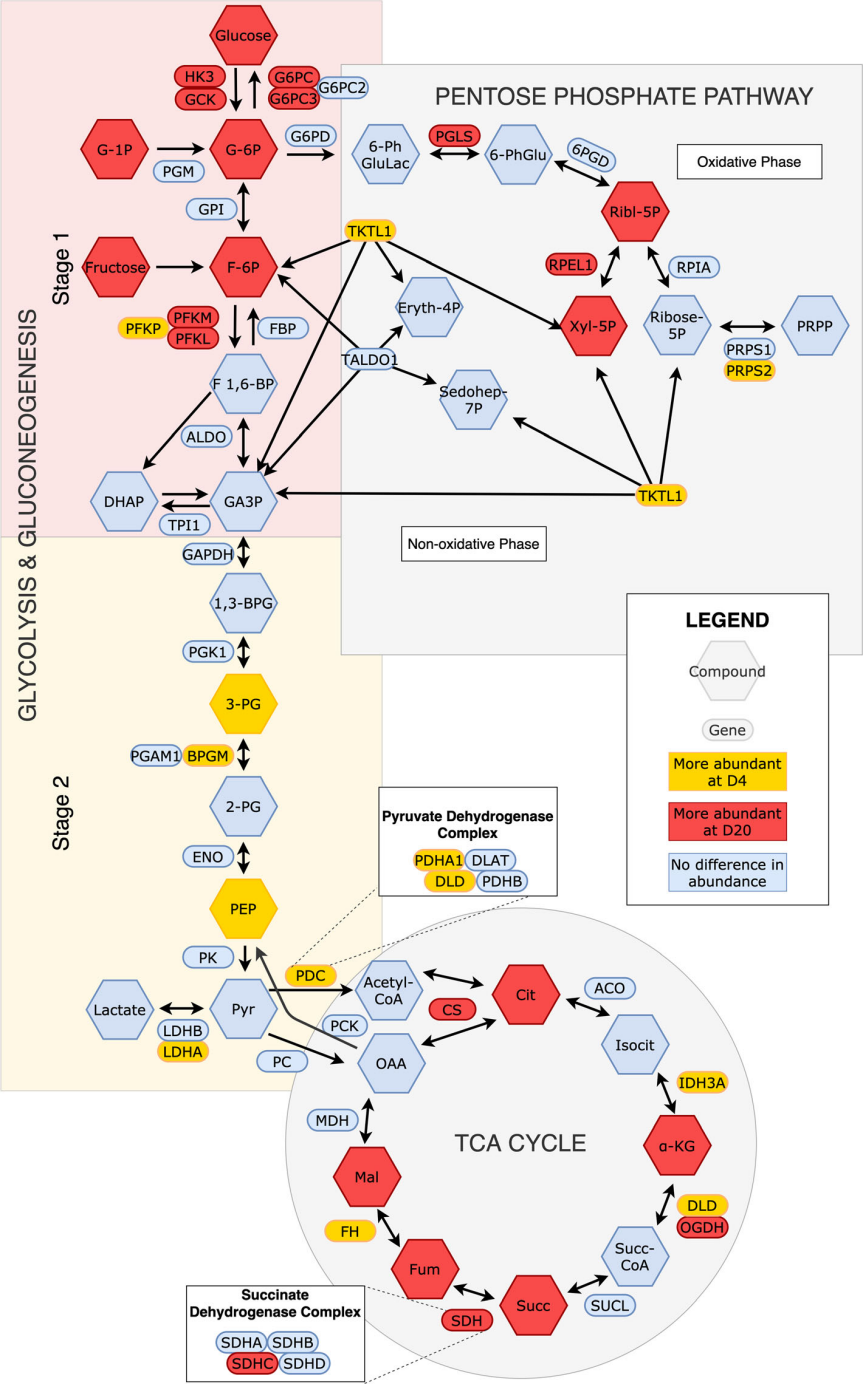
Core carbohydrate metabolism including glycolysis & gluconeogenesis, the TCA cycle, and the pentose phosphate pathway. Genes and metabolites that differed in abundance between days are highlighted, with abbreviations as follows: 1,3-BPG – 1,3-bisphosphoglycerate; 2-PG – 2-phosphoglycerate; 3-PG – 3-phosphoglycerate; 6-PhGluLac – 6-phosphogluconolactone; 6-PhGlu – 6-phosphogluconate; α-KG – α-ketoglutarate; BPGM – bisphosphoglycerate mutase; Cit – citrate; CS – citrate synthase; DHAP –dihydroxyacetone phosphate; DLD - dihydrolipoamide dehydrogenase; Eryth-4P – erythrose-4-phosphate; F-6P – fructose-6-phosphate; F 1,6-BP – fructose-1,6-bisphosphate; Fum – fumarate; G-1P – glucose-1-phosphate; GA3P – glyceraldehyde-3-phosphate; GCK – glucokinase; G6PC – glucose-6-phosphatase catalytic; G6PC3 – glucose-6-phosphatase catalytic subunit 3; G-6P – glucose-6-phosphate; HK3 – hexokinase 3; IDH3A – isocitrate dehydrogenase 3 alpha; Isocit – isocitrate; LDHA – lactate dehydrogenase A; Mal – malate; OAA – oxaloacetate; PDHA1 – pyruvate dehydrogenase E1 subunit alpha 1; PEP – phosphoenolpyruvate; PFKM – phosphofructokinase, muscle; PFKL – phosphofructokinase, liver; PFKP – phosphofructokinase, platelet; PGLS – 6-phosphogluconolactonase; PRPP – phosphoribosyl pyrophosphate; PRPS2 – phosphoribosyl pyrophosphate synthetase 2; Pyr – pyruvate; Ribl-5P – ribulose-5-phosphate; RPEL1 – ribulose-5-phosphate-3-epimerase like 1; Sedohep-7P – sedoheptulose-7-phosphate; SDHC – succinate dehydrogenase complex subunit C; Succ – succinate; Succ-CoA – succinyl-coA; TKTL1 - transketolase like 1; Xyl-5P – xylulose-5-phosphate

Central carbohydrate metabolism consists of glycolysis, gluconeogenesis, the tricarboxylic acid (TCA) cycle, and the pentose phosphate pathway (PPP) (Fig. 6). Glycolysis consists of two stages: 1) Conversion of free glucose to two triose phosphates, 2) energy generation through production of pyruvate. The integrated data suggests that, at D4, the glycolysis pathway is enriched at the second, ATP-generating stage. The transcript encoding one isoform of PFKP, the rate limiting enzyme responsible for conversion of fructose-6-phosphate to fructose-1,6-bisphosphate, was more abundant at D4. This may reflect isozyme selection by HIF1A to increase efficiency of this pathway under hypoxic conditions. Furthermore, two intermediate metabolites (3-PG, PEP), and transcripts encoding two enzymes from the second stage of glycolysis (BPGM, PDHA1) were also enriched in the D4 samples. The enzyme BPGM and metabolite 3-PG represents a branching point in glycolysis. In the glycolysis pathway BPGM acts as a mutase, and regulates the entry of 3-PG into either glycolysis or serine biosynthesis through its effects on PGAM1. The product of BPGM enzymatic activity, 2,3 bisphosphoglycerate (2,3 BPG) serves as a phosphate donor to activate PGAM and promote glycolysis. LDHA, an enzyme involved in anaerobic ATP production, was upregulated at D4, in addition to transporters responsible for both import and export of lactate (SLC16A3, SLC5A12). LDHA favors the conversion of pyruvate to lactate and regenerates the NAD+ required by the glycolytic glyceraldehyde-3-phosphate dehydrogenase (GAPDH). All of these D4 enriched molecules may be critical to supporting production of liver ATP via glycolysis under hypoxic conditions during this early stage post-hatch.

The pyruvate dehydrogenase complex controls the link between glycolysis and the TCA cycle. Transcripts encoding two of the three components of pyruvate dehydrogenase, the E1 subunit (PDHA1) and Dihydrolipoyl dehydrogenase (DLD) were enriched in the D4 liver. In addition, the regulatory kinase PDK1, which inactivates pyruvate dehydrogenase, was also elevated in the D4 samples. The increased abundance of the pyruvate dehydrogenase subunit along with the negative regulatory PDK1 suggests that metabolism at D4 may be primed to respond rapidly to changes in ATP levels and oxygen availability.

Several transcripts encoding rate-limiting sugar kinases involved in the early steps of glycolysis were more abundant at D20 compared with D4 (HK3, GCK, PFKM, PFKL). Corresponding first stage glycolytic metabolites were also more abundant in D20 (glucose, G-6P, F-6P), with G-6P having one of the highest fold changes when compared with D4 (log_2_FC 3.48). HK3 and GCK have key differences in their regulation. GCK specifically acts on glucose, while HK will phosphorylate multiple types of hexoses. GCK also has much lower affinity for glucose than HK, and, unlike HK, GCK is not inhibited by its product, G-6P. Thus, while HK maintains basal glucose metabolism, GCK is responsible for phosphorylating excess glucose for other fates, such as glycogen synthesis or diversion to the pentose phosphate pathway. Phosphofructokinase (PFK) controls glycolytic rate and is under tight control, although there is evidence that isozymes differ in their regulation. Two isoforms of PFK were more abundant at D20 than D4, one of which (liver isoform PFKL) was upregulated in broiler chickens with high growth potential when compared to crosses and layer birds, suggesting that this isoform may contribute to rapid growth rate of maturing birds [29]. The increased abundance of these enzymes and metabolites at D20 suggests surplus of free glucose that can be diverted to other metabolic fates or exported from the liver for use by other tissues.

Glycogen metabolism and gluconeogenesis are two pathways the liver uses to provide glucose to other organs during fasting. Typically, the first resource exploited is glycogen. Glycogen can be synthesized by the enzyme glycogen synthase from glucose-1-phosphate (G-1P) and broken down by glycogen phosphorylase to yield G-1P. Glycogen synthase transcripts along with two isoforms of glycogen phosphorylase (PYGL, PYGB), are enriched in the D20 liver. This, combined with the observation that G-1P is also elevated in the D20 liver, suggests that the D20 liver is capable of rapid response to demands for either glycogen synthesis or phosphorolysis. In addition, the D20 liver is enriched for two glucose-6-phosphatase mRNAs (G6PC, G6PC3), which catalyze the last step of gluconeogenesis. As with glycogen metabolism, it appears that glucose metabolism in the D20 liver is capable of rapid responses to the demands of the body for glucose.

The TCA cycle is an aerobic pathway that continues the oxidation of pyruvate, producing electron donors NADH and FADH_2_ which will go on to oxidative phosphorylation. Multiple components of the TCA cycle are upregulated at D20, indicating greater oxygen availability and abundance of nutrients. At D20, several intermediate metabolites in the TCA cycle were more abundant (citrate, α-ketoglutarate (α-KG), succinate, fumarate, malate), along with mRNAs encoding three enzymes (CS, ODGH, SDHC). All metabolites but α-KG were also among the top most significant compounds at D20, in terms of both log_2_ fold change and significance (see Table 2). α-KG, fumarate, and succinate all serve as entry points for catabolized glucogenic amino acids. CS is the rate-limiting enzyme of the TCA cycle. Elevated citrate is an important regulator of metabolism, with high levels signaling abundant energy. Citrate inhibits glycolysis through its action on phosphofructokinase and stimulates fatty acid synthesis.

Components of the TCA cycle are reduced at D4 compared with D20 livers, consistent with response to hypoxic conditions. Regulation of the pyruvate dehydrogenase complex also suggests metabolic flexibility allowing for rapid response to energy and oxygen levels and utilization of alternative carbon sources for critical metabolites. At D4, four TCA-related transcripts were more abundant (PDHA1, DLD, IDH3A, FH). The rate-limiting pyruvate dehydrogenase complex controls entry of pyruvate into the TCA cycle, and is regulated by several enzymes whose transcripts were also more abundant at D4 (PDP1, PDP2, PDK1). This could represent increased responsiveness of the pyruvate dehydrogenase complex to changes in ATP and oxygen levels. One isozyme of isocitrate dehydrogenase, which interconverts isocitrate and α-KG, was upregulated at D4 (IDH3A). IDH1 and IDH2 can catalyze in both oxidative and reductive directions and are involved in hypoxia response when downregulation of the TCA cycle requires alternate means to synthesize acetyl-CoA and citrate. IDH3A, however, is irreversible and only converts isocitrate to α-KG. IDH3A is also localized to the mitochondria, relies on NAD+ as a cofactor instead of NADP+, and is allosterically regulated by a number of factors. Although hypoxic conditions typically favor conversion of α-KG to isocitrate as an alternative way to generate acetyl-CoA and citrate [30], IDH3A still appears to have a critical role in response to hypoxia. In cancer cells, elevated levels of IDH3A ultimately lead to decreased levels of α-KG. In turn, reduced α-KG levels stabilize the HIF1A protein thereby promoting angiogenesis [31]. Conceivably, the IDH3A mechanism documented in cancer cells may play an important role in the normal development of the early post-hatch liver.

The pentose phosphate pathway utilizes glycolytic intermediates to produce NADPH for reducing power and supplies pentoses for nucleotide synthesis. The non-oxidative branch of the PPP is upregulated at D4, consistent with rapid cell proliferation, while the oxidative branch is upregulated at D20, perhaps to meet increased demand for reducing power. At D4, two transcripts encoding enzymes in the non-oxidative branch of the PPP were upregulated (TKTL1, PRPS2). TKT is the rate-limiting enzyme reversibly linking the PPP with glycolysis. Elevated levels of TKT could indicate intermediates are being exchanged between pathways. The upregulation of PRPS2 suggests that ribose-5-phosphate generated through the non-oxidative branch is going on to purine and pyrimidine metabolism at D4. In contrast at D20, enzymes (PGLS, RPEL1) and metabolites (ribulose-5P, xylulose-5P) involved in the oxidative phase of the PPP were more abundant. Increased levels of RPEL1 suggests that ribulose-5-phosphate is also being recycled back into glycolysis, prioritizing energy production through complete oxidation of G-6P while concurrently producing NADPH to provide the reducing agent needed for lipid synthesis at D20.

#### Amino acid metabolism

**Fig. 7:**
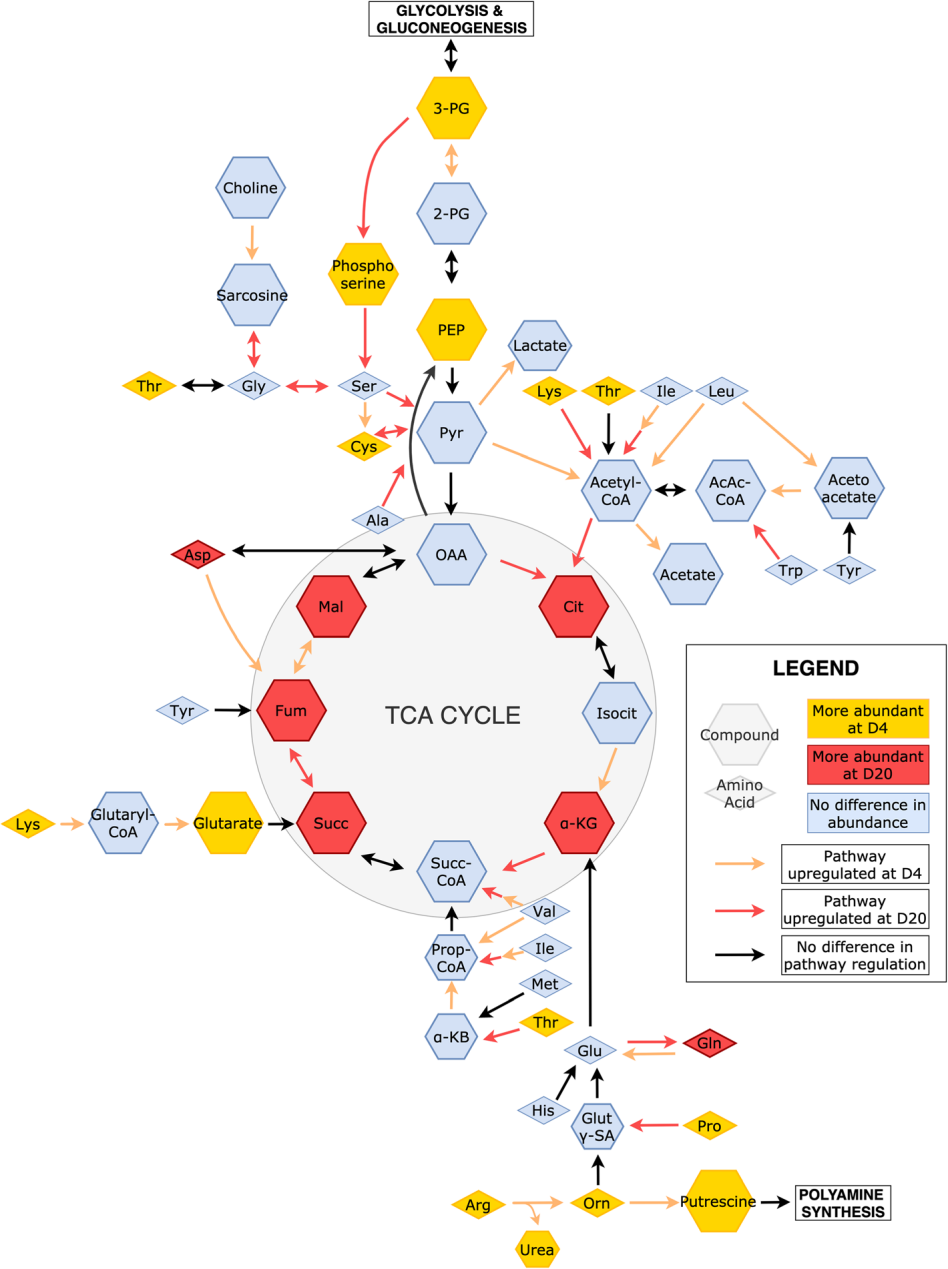
Amino acids as they relate to core metabolic pathways, especially the TCA cycle. Compounds that differed in abundance between days are highlighted, along with colored arrows representing upregulation. Abbreviations are as follows: 2-PG – 2-phosphoglycerate; 3-PG – 3-phosphoglycerate; α-KB – α-ketobutyrate; α-KG – α-ketoglutarate; AcAc-CoA – acetoacetyl-coA; Ala – alanine; Arg – arginine; Asp – aspartate; Cit – citrate; Cys – cysteine; Fum - fumarate; Gln – glutamine; Glu – glutamate; Glut γ-SA – glutamate γ-semialdehyde; Gly – glycine; His – histidine; Ile – isoleucine; Isocit – isocitrate; Leu – leucine; Lys – lysine; Mal – malate; Met – methionine; OAA – oxaloacetate; Orn – ornithine; PEP – phosphoenolpyruvate; Pro – proline; Prop-CoA – propionyl-CoA; Pyr – pyruvate; Ser – serine; Succ – succinate; Succ-CoA – succinyl-coA; Thr – threonine; Trp – tryptophan; Tyr – tyrosine; Val – valine

Amino acids are the building blocks of proteins and also serve many important metabolic functions. Several amino acids, their derivatives, and waste products differed in their abundance between days, including nine more abundant at D4 (arginine, lysine, threonine, cysteine, proline, ornithine, phosphoserine, urea, uric acid) and three more abundant at D20 (aspartate, glutamine, creatinine). Of the amino acids more abundant at D4, three were essential (arginine, lysine, threonine) and three non-essential (cysteine, proline, ornithine). Metabolite data was not able to differentiate ornithine from arginine, so we assume that one or both of them were more abundant at D4. Arginine, ornithine, and proline are glucogenic, typically being converted to glutamate that is readily converted to TCA cycle intermediate α-KG. However, an alternative pathway allows glutamate to be converted to succinate. Cysteine is glucogenic and can be converted to pyruvate. Lysine was one of the top most significant metabolites more abundant at D4 and is ketogenic through acetyl-CoA. Threonine is both glucogenic, through succinyl-CoA, and ketogenic, through acetyl-CoA. Phosphoserine is an intermediate between glycolysis and serine production. Urea and uric acid are both nitrogenous waste products. At D20, both amino acids that were more abundant were non-essential and glucogenic (glutamine, aspartate). Glutamine is converted to glutamate, while aspartate is converted to oxaloacetate. These differences in abundance may reflect increased catabolism of amino acids at D20, or differences in utilization of amino acids between days (Fig. 7).

As discussed above, at D4, the transcriptome data indicates that BPGM is shunting the intermediate 3-PG is towards glycolysis. In contrast at D20, the downregulation of BPGM suggests glycolytic intermediates are being directed towards serine biosynthesis. Two other transcripts encoding enzymes related to serine biosynthesis from glycolytic intermediates were upregulated at D20 (PHGDH, GLYCTK). PHGDH directs 3-PG towards serine biosynthesis, while GLYCTK converts glycerate to glycolytic intermediate 2-PG, a precursor of 3-PG. Several transcripts encoding enzymes involved in serine and glycine metabolism were also upregulated at D20 (SDSL, AGXT, PIPOX, SARDH, GNMT, ALAS2, GCAT, AOC3). AGXT catalyzes a number of reactions, including the interconversion of serine and glycine, interconversion of serine and hydroxypyruvate, and interconversion of glycine and glyoxylate. Both hydroxypyruvate and glyoxylate can go into glyoxylate metabolism. Although the main enzymes of the glyoxylate cycle have not been found in chickens, the liver has been observed to have glyoxylate activity [32]. SARDH and PIPOX generate glycine from sarcosine, while GNMT interconverts sarcosine and glycine. Sarcosine is an intermediate between glycine, creatine, and choline metabolism. SDSL catabolizes serine to pyruvate and also converts threonine to 2-oxobutanoate, an alpha-ketoacid intermediate of threonine catabolism, to succinyl-CoA. ALAS2, GCAT, and AOC3 are all involved in generating different metabolites from glycine.

Proline and lysine metabolism may indicate increased collagen production and remodeling at D20. Although both metabolites were more abundant at D4, several enzymes facilitating their incorporation into collagen were upregulated at D20, (PYCR1, PYCRL, P4HA2, LOC425607, L3HYPDH, HYKK). PYCR1 and PYCRL are involved in the interconversion of proline, hydroxyproline, and pyrroline-5-carboxylate. P4HA2 and LOC425607 are involved in formation of collagen structural components from 4-hydroxyproline or hydroxylysine, respectively. HYKK is a kinase that phosphorylates hydroxylysine residues. One enzyme involved in collagen synthesis was upregulated at D4 (PLOD2), which is responsible for hydroxylation of lysine residues, allowing for cross-linking and stabilization of collagen.

Several transcripts upregulated at D4 encode enzymes that yield alternative TCA cycle intermediates, while several transcripts upregulated at D20 encode enzymes generating pyruvate from amino acids. In lysine degradation, two metabolites (lysine, glutarate) and two enzymes (DLD, DHTKD1) were more abundant at D4. DLD and DHTKD1 convert 2-oxoadipate to glutaryl-CoA, which can then be converted to glutarate and enter the TCA cycle through succinate. In contrast, EHHADH was upregulated at D20, supporting the canonical pathway of lysine degradation to acetyl-CoA. At D4, mRNAs encoding enzymes affecting aspartate and glutamate (ADSSL1, ALDH5A1) were enriched. ADSSL1 converts aspartate to fumarate while ALDH5A1 metabolizes glutamate to succinate. Under normoxic conditions, aspartate is converted to oxaloacetate and glutamate is converted to α-KG. Given the TCA cycle is downregulated at D4 due to hypoxia, diverting these amino acids to different fates may allow them to be utilized more efficiently. Furthermore, this may serve a regulatory role in controlling levels of α-KG. Hence, the D4 liver may be relying on amino acids that are metabolized to intermediates suitable for anaerobic energy. This is further supported by the only TCA-specific mRNA upregulated at D4 encodes FH. Enrichment for FH would allow regeneration of oxaloacetate, which could then be converted to PEP, feeding directly into pyruvate for lactate production. At D20, mRNAs encoding enzymes producing pyruvate from various amino acids were upregulated (SDSL, CCBL1, AGXT). SDSL can convert serine to pyruvate while CCBL1 can interconvert cysteine and pyruvate. AGXT can convert alanine to pyruvate as part of the Cahill cycle, either replenishing levels of blood glucose through gluconeogenesis or oxidizing pyruvate for energy production in the TCA cycle and oxidative phosphorylation. Consequently, the liver at D20 appears to be utilizing amino acids for energy through canonical degradation pathways.

Homocysteine metabolism shows different directionality between days, possibly favoring the synthesis of cysteine at D4 and the synthesis of methionine at D20. Cysteine is synthesized from cystathionine, made from conjugating serine with methionine-derived homocysteine. The mRNA encoding the enzyme producing cystathionine was upregulated at D4 (LOC418544), along with metabolites cysteine and 2-hydroxybutanoic acid. 2-Hydroxybutanoic acid was one of the top most significant metabolites at D4 (Table 2), and is a byproduct of cysteine production. It is elevated when cysteine is limiting, such as when oxidative stress or detoxification needs are high. At D4, higher levels of cysteine may be required to combat oxidative stress. In contrast at D20, two enzymes contributing to homocysteine production are upregulated (CCBL1, AHCYL1), suggesting that homocysteine is shunted to methionine salvage. Methionine is essential and one of the key amino acids important for growth in chickens [33, 34].

Degradation of the branched chain amino acids valine, leucine, and isoleucine may serve different purposes between time points: generation of branched chain fatty acid precursors at D4, and complete catalysis for energy production at D20. Valine is glucogenic, entering the TCA cycle through degradation to succinyl-CoA. Leucine is ketogenic, as its metabolism results in acetyl-CoA. Isoleucine can follow either glucogenic or ketogenic routes. Seven enzymes involved in branched chain amino acid metabolism were more abundant at D4 (BCAT1, DLD, BCKDHB, ALDH6A1, AUH, AACS, HIBADH) than D20. BCAT1, DLD, and BCKDHB are involved in the early steps of degrading these branched chain amino acids. HIBADH and ALDH6A1 are involved in valine catabolism. These enzymes are responsible for the catabolism of valine to propanoyl-CoA. AUH and AACS are involved in leucine metabolism yielding acetoacetyl-CoA. As liver cannot efficiently metabolize acetoacetyl-CoA [35], it is exported for use by other organs. Four mRNAs encoding enzymes impacting branched-chain amino acids were enriched at D20 (EHHADH, HSD17B10, ACSS2, ACSS1L). HSD17B10 and EHHADH are dehydrogenases involved in beta-oxidation of a variety of compounds. HSD17B10 is mitochondrial and oxidizes steroids, while EHHADH is a bifunctional enzyme localized to the peroxisome and participates in multiple steps of branched-chain amino acid degradation as well as fatty acid oxidation. ACSS2 and ACSS1L activate short-chain fatty acids for further metabolism, preferring acetate but also acting on propionate ultimately giving rise to citrate.

Arginine appears to contribute to polyamine synthesis at D4, consistent with increased cell proliferation. Four metabolites (urea, putrescine, ornithine, arginine) and transcripts encoding enzymes (ARG2, ODC1, GATM, AMD1, CHDH, DMGDH) involved in this were more abundant at D4. ARG2 converts arginine to ornithine, releasing urea as a byproduct. ODC1 converts ornithine to putrescine, a polyamine critical for cell proliferation [36]. GATM supports the conversion of glycine to arginine through guanidinoacetate. CHDH and DMGDH support the catabolism of choline to sarcosine, a glycine precursor, which could provide additional substrates for polyamine synthesis. AMD1 is involved in methionine salvage, where is generates S-adenosyl-methionine (SAM), an important methyl group donor and component of polyamine synthesis. In addition to contributing to cell proliferation, this pathway may also divert excess arginine away from α-KG. The increased breakdown of arginine for polyamine synthesis could also explain the increased abundance of urea at D4. In contrast, one enzyme converting arginine to agmatine (AZIN2) was upregulated at D20, signifying a possible alternate route to polyamine synthesis.

Transcriptome data suggests that glutamate and glutamine metabolism is reversed between D4 and D20. Metabolism favoring glutamine production appears favored at D20, while at D4, glutamine conversion to glutamate is likely enhanced. Glutamate-ammonia ligase (GLUL, elevated at D20) synthesizes glutamine from glutamate and ammonia, while glutaminase (GLS, elevated at D4) converts glutamine to glutamate and ammonia. In adult birds, the synthesis of uric acid, the predominate nitrogen waste product, requires glutamine. In contrast, enrichment for glutamate at D4 can support production of succinate, which has important roles in stabilizing the hypoxia response [37].

#### Lipid metabolism

At hatch, chicks are supplied with lipids absorbed from the yolk sac while in mature birds the liver receives dietary lipids directly from the small intestine via the hepatic portal vein. Yolk nutrients serve as the main source of lipids for D4 birds, as bile acid capabilities to facilitate dietary lipid absorption are not fully developed until after the first week post-hatch [38, 39]. Post-hatch, the yolk sac membrane is able to continue absorbing lipids directly into the bloodstream through endocytosis, bypassing the small intestine and the need for liver-produced bile acids; this lipid reservoir provides fuel for early post-hatch growth [38]. Compared with D20 liver, the D4 liver metabolome evidences enrichment of multiple yolk-derived fatty acid metabolites including: palmitoleic, myristic, linoleic, and oleic acid. Two acyl synthetases involved in the activation of fatty acids were upregulated at D4 (ACSL3, ACSL4), Acyl synthetases activate free fatty acids in the cytoplasm via conjugation with an acyl group. ACSL3 preferentially acts on myristic acid, arachidonic acid, lauric acid, and eicosapentaenoic acid, and is involved in mediating hepatic lipogenesis. ACSL4 prefers arachidonic acid and is often expressed in steroid-producing organs. Two enzymes (CPT2 and ACAD9) involved in fatty acid beta-oxidation were upregulated at D4, and appear representative of the available substrates in yolk-derived lipids at D4. CPT2 prepares activated fatty acyl-CoAs for oxidation by removing the acyl group once they have entered the mitochondria. ACAD9 is a mitochondrial acyl-CoA dehydrogenase catalyzing the rate-limiting step of beta-oxidation, with a preference for palmitoyl-CoA and long-chain unsaturated fatty acids. The substrate preferences of ACAD9, ACSL3 and ACSL4 are consistent with an abundance of yolk-derived fatty acids consisting of mainly palmitate and myristate.

Several enzymes involved in lipid elongation in the endoplasmic reticulum (ELOVL7, HACD2, HACD3) were upregulated at D4, suggesting the synthesis of lipids meeting different requirements such as cell membrane components. Elongation in the ER gives rise to long-chain and very-long-chain fatty acids (>C16). ELOVL7 catalyzes the rate-limiting first step, preferring long-chain and very-long-chain fatty acids (C18, C16, C20), and is involved in synthesizing membrane lipid precursors and lipid mediators. HACD2 and HACD3 are dehydratases involved in the dehydration step of lipid elongation. These long chain fatty acid products could be used within the liver or exported to other tissues to support the rapid growth seen in chicks at D4. Two enzymes associated with mitochondrial fatty acid biosynthesis were upregulated at D20 (MCAT, MECR). Enzymes acting as part of the fatty acid synthase type II (FAS2) complex catalyze mitochondrial fatty acid synthesis. Malonyl transacylase (MCAT), catalyzes the transfer of malonyl from malonyl-CoA to a scaffold protein, beginning the elongation phase. Enoyl ACP reductase (MECR) catalyzes the final step with palmitate as the main product of fatty acid synthesis in the mitochondria.

Transcripts encoding enzymes involved in beta-oxidation in the mitochondria and peroxisome were more abundant at D20 including: EHHADH, ECI1, DECR2, and ACAD11. While mitochondrial beta-oxidation generates acetyl-CoA for energy production, peroxisomal beta-oxidation breaks down very long chain fatty acids (>22C), branched fatty acids and leukotrienes to acetyl-CoA and medium chain fatty acids for biosynthesis of specific fatty acids. EHHADH is a multi-functional, peroxisomal enzyme that acts as both an enoyl-CoA hydratase and a 3-hydroxyacyl-CoA dehydrogenase, catalyzing multiple steps in beta-oxidation. Along with EHHADH, ECI1 rearranges double bonds in unsaturated fatty acids to facilitate their oxidation. DECR2 is a peroxisomal enzyme degrading varying lengths of unsaturated fatty acids. ACAD11 encodes mitochondrial acyl-CoA dehydrogenase catalyzing beta-oxidation of fatty acids with chain lengths of 20 to 26 carbons.

Four thioesterase transcripts were enriched at D20 (ACOT4, ACOT8, ACOT11, THEM4) while only one (ACOT12) was enriched at D4. Thioesterases hydrolyze acyl-CoA to coenzyme A and free fatty acids. ACOT4 is peroxisomal and acts on succinyl-CoA, glutaryl-CoA, and long chain saturated acyl-CoAs. ACOT8 is peroxisomal and has the least specific substrate preferences, acting on medium chain (C2 to C20) saturated or unsaturated fatty acyl-CoAs and also bile acids. ACOT11 prefers long chain substrates including palmitoyl-CoA and myristoyl-CoA, which can go on to mitochondrial beta-oxidation. THEM4 is mitochondrial and prefers medium to long chain substrates (C14 to C18). ACOT12, the only thioesterase upregulated at D4, prefers acetyl-CoA as a substrate.

In glycerolipid metabolism, metabolites and gene expression patterns indicated triacylglycerol (TAG) breakdown was favored at D4 while TAG synthesis was favored at D20. Two metabolites (3-PG, 1-monopalmitin) and several enzymes (LIPC, LIPG, AKR1B10L2, PLPP1, LPIN1, GPAM) were more abundant at D4. LIPG (endothelial) and LIPC (hepatic) are lipases that hydrolyze the ester bond of TAGs to form monoacylglycerol and free fatty acids. 1-Monopalmitin was one of the top metabolites contributing to random forest classification at D4. At D20, one metabolite (glycerol-3-phosphate) and several enzymes (DGKA, DGKZ, DGAT2, AWAT1, TKFC, GLYCTK, GPAT4, GPAT2, AGPAT4, AKR1B10L1) were more abundant. Glycerol-3-phosphate provides the glycerol backbone for TAG synthesis. Three acyltransferases (AGPAT4, GPAT2, GPAT4) are involved in different steps generating phosphatidate from glycerol and acyl-coA, and two acyltransferases (DGAT2, AWAT1) are responsible for the final step of TAG synthesis. At D4, a different acyltransferase involved in generating phosphatidate from glycerol and acyl-CoA was upregulated (GPAM). GPAM is a mitochondrial acyltransferase that prefers saturated fatty acids. In the preparation or recycling of the glycerol backbone, one aldo-keto reductase catalyzing the interconversion of glyceraldehyde and glycerol was upregulated at D4 (AKR1B10L2) while one was upregulated at D20 (AKR1B10L1), and one kinase converting glycerate to glycolytic intermediate 2-PG was upregulated at D20. The interconversion of phosphatidate and diacylglycerol shows contrasting directionality between days; two phosphatases that catalyze the conversion of phosphatidate to a diacylglycerol were upregulated at D4 (PLPP1, LPIN1), while two kinases phosphorylating diacylglycerols to phosphatidate (DGKA, DGKZ) were upregulated at D20. Given the importance of phosphatidate as an important precursor molecule for both TAG synthesis and membrane lipid synthesis, this may represent an important metabolic reprogramming between time periods.

## Discussion

**Fig. 8:**
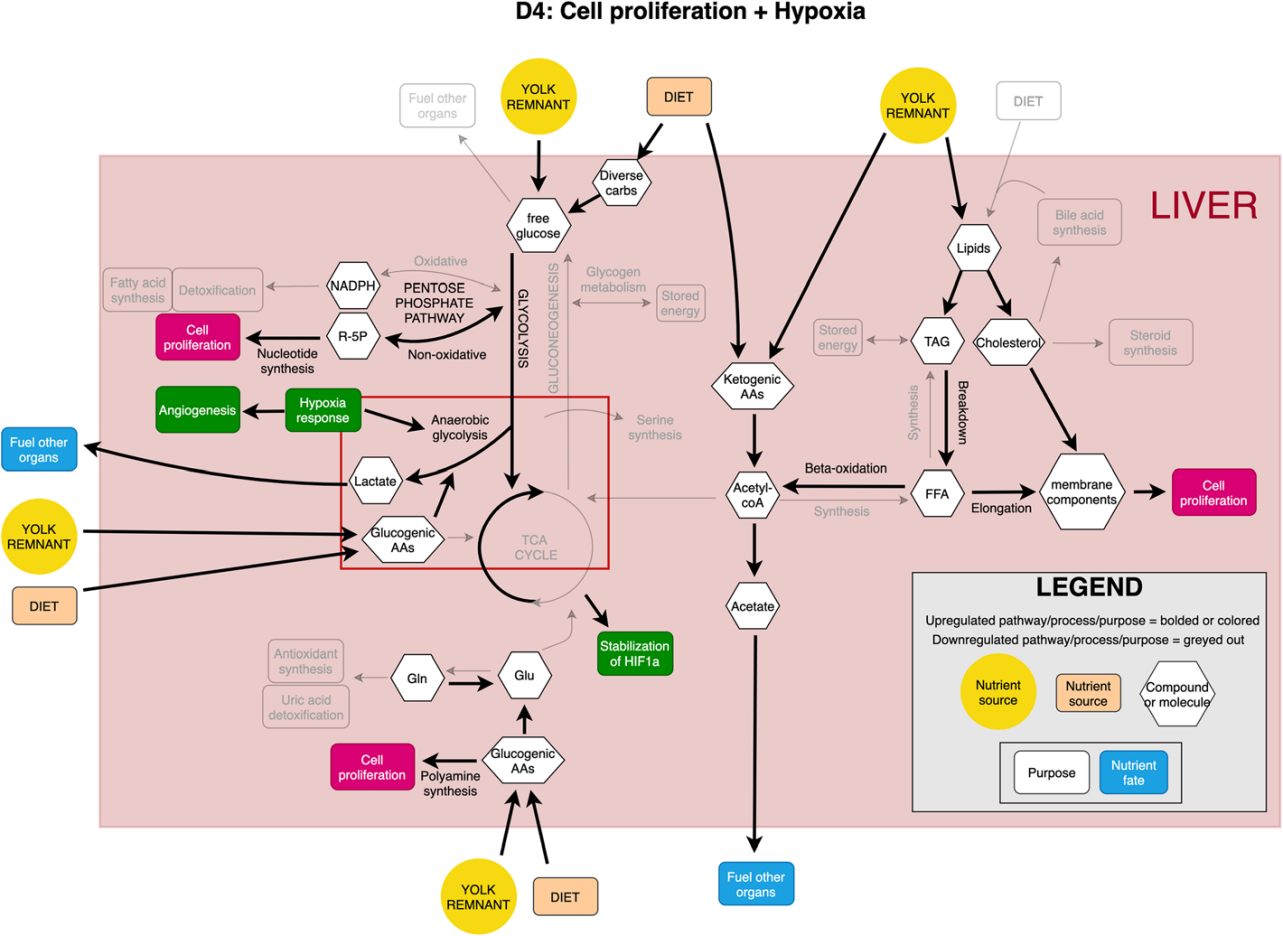
Summary of metabolic reprogramming at D4, in terms of core metabolic pathway regions and biological processes that are upregulated or important. Upregulated components are in color, downregulated components are greyed out. Note: Some arrows represent pathways involving multiple steps

**Fig. 9:**
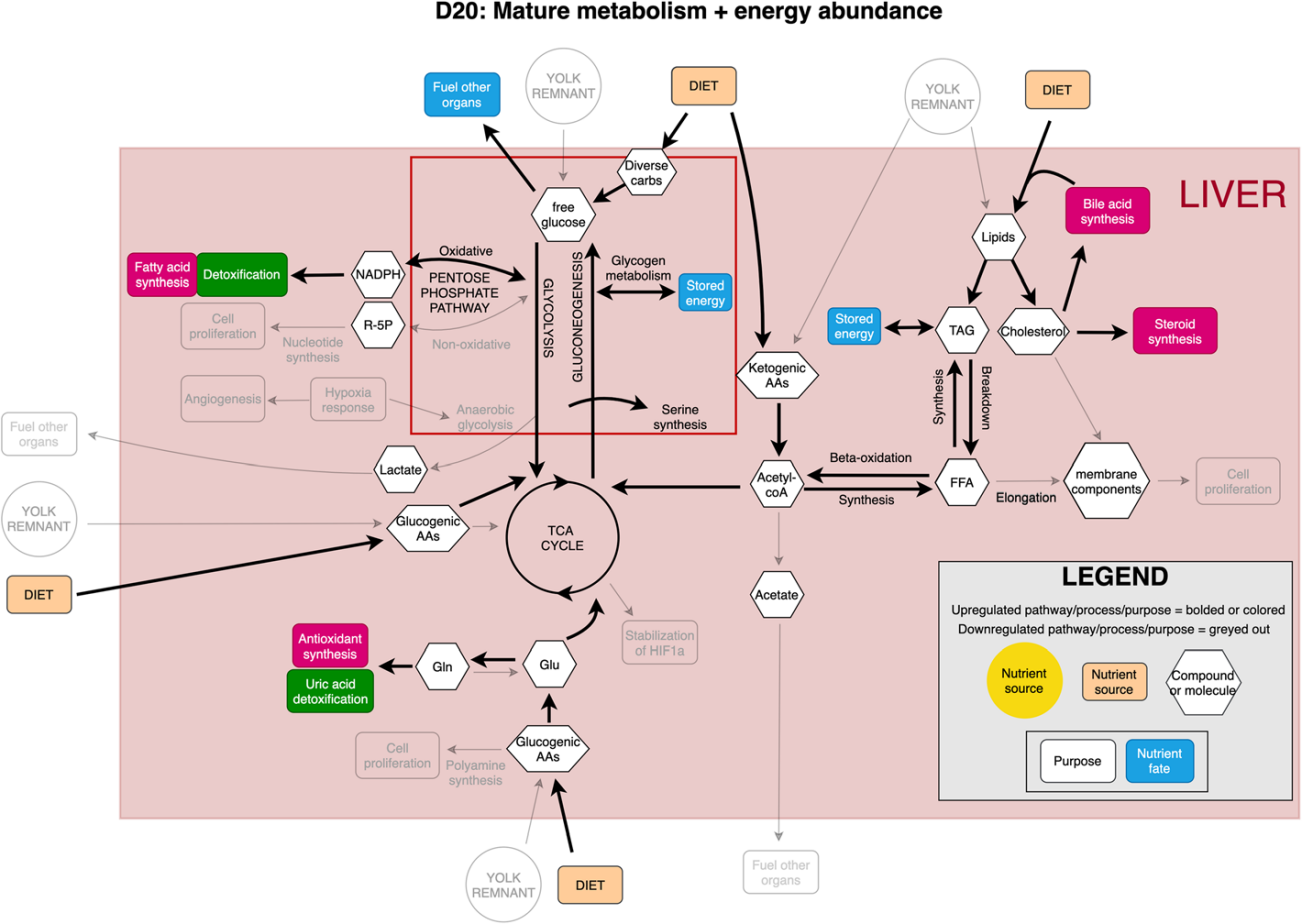
Summary of metabolic reprogramming at D20, in terms of core metabolic pathway regions and biological processes that are upregulated or important. Upregulated components are in color, downregulated components are greyed out. Note: Some arrows represent pathways involving multiple steps

Modern broilers are the product of intensive selection for rapid growth, increased skeletal muscle production, and improved feed efficiency. The liver, as the metabolic center of the body, plays an important role in supporting the emergence of these traits. This study indicates that the significant metabolic reprogramming of the liver occurs between D4 and D20 post-hatch, summarized in Figs. 8 and 9, respectively. This reprogramming is choreographed by modulation of gene expression patterns responding to changes in the proliferative needs of the liver and other tissues. In addition, the nutritional sources of the birds change during this time, shifting from stored yolk to oral nutrition. At D4, liver cells are undergoing rapid proliferation as indicated by the positive allometric growth of the organ at this time. This is supported by the transcriptome analysis that identified enrichment for multiple genes involved in cell proliferation, cell cycle, and DNA replication at D4. In addition, the integrated transcriptome and metabolome data indicates metabolic flexibility especially in carbohydrate metabolism. In glycolysis, D4 liver is manifesting metabolism reminiscent of the Warburg effect, which was first noted in rapidly dividing cancer cells [40, 41] and has been shown to be common in normal proliferating cells [42]. In contrast, by D20 the liver’s relative growth has slowed and the transcriptome has shifted from supporting cell division to enrichment for immune function. Metabolomic data indicates D20 liver no longer displays upregulated anaerobic energy production in carbohydrate metabolism and has taken on the role of acting to store glucose and synthesize fatty acids, functions associated with the mature liver.

An important hypothesis arising from this work is that the D4 liver is hypoxic, which may arise from vascularization lagging behind the rapid growth of the liver. Enrichment of the hypoxia responsive transcription factor HIF1A transcript seen at D4 could lead to increased expression of angiogenic growth factor receptors (VEGF receptors KDR and FLT1) to improve vascular development. HIF1A also plays a central role in increasing glycolytic rate through isozyme selection, and by triggering the Warburg effect; with a central impact of increasing expression of the LDHA isoform that directs glycolysis towards lactate production [43, 44]. The NAD^+^ produced from the reduction of pyruvate to lactate can support the GAPDH enzymatic activity required to drive the ATP production phase of glycolysis. This is necessary to rapidly dividing cells under hypoxia as it maintains ATP production. In addition, lactate secreted from the cell could reduce the blood pH at D4 compared with D20 (Table 1). The reduced pH that results from lactate may further promote matrix rearrangement and angiogenesis [45]. Isozyme selection by HIF1A at D4 is also evident in upregulation of platelet-type phosphofructokinase (PFKP). Metabolic reprogramming similar to that observed in cancer, driven by HIF1A, likely allows D4 liver to continue efficient energy production under hypoxia, while increasing angiogenesis to eventually escape these conditions as the chick matures.

The data indicates that pyruvate occupies an important metabolic fork in the D4 liver. Along with PDK, transcriptome data indicates that two components of the PDH complex, and two pyruvate dehydrogenase phosphatases (PDP) are enriched in the D4 liver. This suggests that pyruvate metabolism in the early post-hatch liver exhibits greater metabolic flexibility than is seen in a cancer cell exhibiting the Warburg effect. The growing liver tissue at D4 retains the option of reverting to aerobic glycolysis and the TCA cycle conferred by tight regulation of this metabolic fork. In turn, this allows for a rapid shift between pyruvate being converted to either lactate in the cytoplasm or acetyl-CoA and then citrate in the mitochondria as metabolic demands change during growth.

In the hypoxic conditions at D4, where glycolysis is the main ATP-producing pathway, metabolic flexibility also allows for differences in nutrient utilization. We hypothesize that the liver is conserving glycolytic intermediates for energy production, including glucogenic amino acids and recycled glycerol backbones, while sparing free glucose for the rest of the body. This is supported by the overall downregulation of the early stages of glycolysis and gluconeogenesis at D4, along with multiple examples of glucogenic amino acids being directed towards oxaloacetate or pyruvate precursors for anaerobic glycolysis. Transcripts encoding enzymes involved in degradation of ketogenic amino acids are upregulated at D4, along with those converting acetyl-CoA to acetate and acetoacetate to acetoacetyl-CoA. The resulting lactate, ketones, short chain fatty acids and other compounds may act as important sources of energy for other tissues. For example, free acetate produced in the liver can be exported for use by other tissues such as cardiac muscle [46], while skeletal muscle can oxidize lactate [47]. The dramatically increased expression of two monocarboxylate transporters – SLC16A3 (log_2_FC 3.29) and SLC5A12 (log_2_ 5.22) – at D4 support the increased import and export activity of compounds like lactate, pyruvate, and oxo acid products of branched chain amino acid catabolism, further suggesting that differences exist in the metabolic demand for these compounds between time points, in both the liver and rest of the body.

Inspection of glycolysis metabolites suggests a second metabolic fork is present in this pathway at 3-phosphoglycerate. BPGM, the enzyme controlling this fork, is upregulated at D4. Knockout of BPGM in mice increases serine and phosphoserine biosynthesis [48]. Thus, elevated BPGM levels at D4 suggest that it is directing 3-PG towards glycolysis rather than serine biosynthesis. In addition to modulating this branching point, BPGM is known for its role in decreasing hemoglobin’s affinity for oxygen. The metabolite 2,3-BPG which is the product of BPGM enzymatic activity, can escape cells and promote the release of oxygen from red blood cell hemoglobin, possibly ameliorating anoxic conditions [49]. Both 3-phosphoglycerate and phosphoserine are enriched in the D4 liver suggesting that some of the glycolytic intermediates are exchanged with serine production at this time. Serine serves several important functions in the metabolism of rapidly growing cells [50], including its roles in the synthesis of proteins, phospholipids, cysteine, glycine and single carbon metabolism. Phospholipids are incorporated into the membranes and single carbon metabolism is essential to the formation of many different metabolites.

The origin of the 3-phosphoglycerate fork may be explained by the enrichment for phosphoenolpyruvate at D4. Pyruvate kinase (PKLR) in the chicken is orthologous to human PKM1/2. In humans, proliferating cells frequently express the embryonic form of pyruvate kinase, which is responsible for the formation of pyruvate from phosphoenolpyruvate [51]. The activity of PKM2 is responsive to allosteric regulators and post-translational modifications. In proliferating cells, PKM2 activity is inhibited, yielding elevated levels of phosphoenolpyruvate (seen at D4) and other glycolytic products [52]. Some of these products may be diverted from pyruvate production to anabolic processes such as serine production or supporting nucleotide production via the non-oxidative phase of the pentose phosphate pathway (PPP). Elevated levels of the TKT1 and PRPS2 transcripts in the D4 liver support enrichment for the non-oxidative PPP phase. Alternative splicing of the PKM transcript produces PKM2. Based on what is known about PKM1/2 in humans, we hypothesize that in the chicken, PKLR is similar in both role and splicing. Our current analysis does not distinguish splice products, so investigation of the hypothesis of the potential role of PKLR in D4 glycolytic regulation awaits future work.

Consistent with a hypoxic environment, metabolomic data indicates that the TCA cycle is suppressed in the D4 liver compared with D20. However, transcripts encoding enzymes functioning in the TCA cycle including Isocitrate Dehydrogenase 3A (IDH3A), Dihydrolipoamide Dehydrogenase (DLD), and Fumarate Hydrolase (FH) are enriched at D4. Elevated IDH3A and DLD protein expression would reduce levels of α-ketoglutarate, a potent inhibitor of HIF1A activity [31]. FH could function in the anaplerotic roles of glutamate, lysine, threonine and proline at D4. These amino acids would replenish the levels of oxaloacetate that is needed to react with acetyl-CoA to yield mitochondrial citrate. This mechanism is apparently seen when citrate is being actively transported from the mitochondria to support lipid synthesis [53]. In addition, glutamate and threonine can be metabolized to Succinyl-CoA, an important precursor along with glycine to the synthesis of porphyrins.

Another metabolic pathway enriched at D4 is the synthesis of polyamines, which are important to sustaining proliferation. This pathway can also explain the elevated levels of urea seen in the D4 chick. While urea is the typical excretory product of nitrogen metabolism in mammals, very little is produced in the liver of chickens with most nitrogen waste disposed of as uric acid. However, urea is produced as a byproduct of an early step in the synthesis of polyamines, the enzymatic conversion of arginine to ornithine by arginase (ARG2). Hence, we hypothesize that this is the origin of the elevated urea in the D4 compared with the D20 liver.

By D20, the liver is apparently under normoxic conditions with the expression of HIF1A reduced compared to D4. TCA enzymes, mitochondrial processes including oxidative phosphorylation, and other oxygen-dependent metabolic pathways are also more active at D20. Comparison between the D20 and D4 transcriptome and metabolome provide evidence for significant metabolic reprogramming between the 2 days. By D20, glucose metabolism is enriched for transcripts and metabolites associated with phase 1 of the glycolysis pathway, with glucose-6-phosphate residing at a metabolic fork. Transcripts encoding enzymes associated with both glycolysis (HK3, GCK, PFKL, PFKM) and gluconeogenesis (G6PC and G6PC3) are enriched at D20. This is consistent with the need of the mature liver to capture glucose by phosphorylation, or export glucose during fasting for use by other tissues. The G6P can be converted to glucose 1-phosphate (enriched at D20) and stored as glycogen. Alternatively, G6P can be converted to 6-phosphogluconolactone, which is the first step of the pentose phosphate pathway. In fact, by D20 the pentose phosphate pathway has become enriched for components of the oxidative phase, likely increasing the production of NADPH to support the need for fatty acid synthesis and detoxification of compounds. Upregulation of glycolytic enzyme PFKL has also been linked to growth potential in broilers, with one study suggesting that this isoform may contribute to the rapid growth rate of maturing birds [29].

In contrast to D4, TCA cycle metabolites are elevated at D20, possibly by translation of the enriched citrate synthase transcript. We hypothesize that the elevated levels of citrate play a fundamental role in the metabolic reprogramming seen between D4 and D20. Citrate plays multiple roles as a metabolite and as a regulator of enzymatic activities. As a metabolite, it is the first component of the mitochondrial TCA cycle and can be exported to the cytoplasm and metabolized to support both nucleotide and fatty acid synthesis. As an enzyme regulator it serves as an indication of the energy state of the cell, becoming abundant at high ATP levels. Citrate inhibits the activity of phosphofructokinase (PFK) preventing the ATP synthesis phase of glycolysis, as is suggested in the D20 data. Citrate also serves as an activator of acetyl-CoA-carboxylase (ACACA) [54], the first enzyme in the cytoplasmic synthesis of fatty acids. Although there was no measurable difference between D4 and D20 ACACA mRNA levels, high levels of citrate would likely drive fatty acid synthesis in the D20 liver. Another impact of a high citrate level that results in elevated malonyl-CoA is inhibition of CPT2 that is part of the lipid transport system that brings fats into the mitochondria for beta-oxidation.

D20 transcriptome and metabolome data provide additional information relevant to fatty acid metabolism. Transcripts encoding enzymes driving mitochondrial fatty acid synthesis and those involved in beta-oxidation in both the mitochondria and peroxisome were elevated at D20 compared with D4. Peroxisomal beta-oxidation is a major source of acetate that can be released from the liver for use by other tissues following conversion to acetyl-CoA [55, 56]. The D20 liver is enriched in glycerol-3-phosphate, an important metabolite in triacylglycerol synthesis. The potential importance of triacylglycerol synthesis in the D20 liver is also supported by enrichment for transcripts encoding enzymes responsible for phosphatidate synthesis, which is essential for TAG production. Also enriched were transcripts encoding phosphatidylcholine synthetic enzymes. In addition to its role in lipid membranes, phosphatidylcholine is a major component of very low-density lipoproteins, which are secreted from the liver to transport TAGs.

## Conclusions

The liver of the modern broiler must meet the needs of rapid growth that has resulted from intensive genetic selection. Allometric comparisons indicate that the modern broiler liver reaches its maximum normalized size several days earlier than slower growing chickens. As slow growing and modern broilers are of similar size at hatch, this suggests that selection has compressed the positive allometric growth phase of the liver into the first week post-hatch. The transcriptome and metabolome of the D4 liver indicate the tissue is undergoing rapid proliferation under hypoxic conditions with carbohydrate, lipid and amino acid metabolism primed to support this growth. At D4, ATP is likely to be largely derived from glycolysis, the PPP is favoring production of nucleotide precursors and returning carbon backbones to glycolysis, and lipid biosynthesis appears to support fatty acid elongation, perhaps for membrane production in the proliferating cells. By D20, the liver is undergoing negative allometric growth and has transitioned from predominantly supporting its own proliferation, to supporting the metabolic needs of other tissues. Metabolic reprograming has shifted the glycolytic pathway to the ATP investment phase allowing for rapid responses to either store or release glucose, the PPP now appears to be shifted towards producing NADPH to support anabolic reactions such as lipid production and lipid metabolism appears to have shifted to triacylglycerol production for lipid export.

Understanding the molecular and physiological changes occurring in post-hatch broiler liver by exploiting high-throughput methods has broad utility. A major research goal at the intersection of computation and biology involves understanding the strengths and limitations of different types of data, as well as the relationship between them. In any system, comparing knowledge gained from different types of high-throughput technologies can corroborate existing biology and generate new, testable hypotheses. Lastly, the chicken is a valuable, accessible, and well-supported model both in terms of research and infrastructure for studying the effects of genetic selection especially as it relates to metabolic efficiency. In this work we have developed a preliminary contrast of the liver at two time points representing critical biological differences in the modern broiler. Some of our observations recapitulate known biology and provides possible explanations for phenotypic characteristics and allometric relationships observed in the modern broiler. While hypoxia has a documented role in controlling embryonic development [57], the role of hypoxia in normal post-hatch or post-natal tissue development is not well characterized. A major future goal will be to build networks of interactions to better understand the regulation of this metabolic reprogramming as the growing chicken transitions from hypoxic to normoxic conditions.

## Methods

### Bird husbandry, necropsy, and tissue collection

Day-old male Ross 708 chicks were obtained from a commercial hatchery (Mountaire Farms, Millsboro, DE) and grown on the University of Delaware Farm (Newark, DE). Standard management and husbandry procedures were followed, as approved by the Animal Care and Use Committee of the University of Delaware (IACUC 72R-2017-0). All experiments were performed in accordance with relevant guidelines and regulations. On each even-numbered day post-hatch from Day 4 (D4) through Day 20 (D20), 12 birds were randomly chosen and humanely euthanized by cervical dislocation. Prior to euthanasia, birds were weighed and blood was drawn from the brachial wing vein for immediate i-STAT blood chemistry analysis using CG8+ cartridges. 200 uL of blood was also collected in EDTA tubes on ice and then centrifuged to separate plasma for metabolome analysis. During necropsy, multiple tissues were systematically collected and snap frozen in liquid nitrogen. Select organ masses (heart, liver, breast muscle) and intestine segment lengths were also recorded. Liver was collected from the caudal portion of the left lobe, with an additional tube of tissue saved for metabolome analysis. Frozen tissues and plasma were subsequently stored at -80 °F until further use. Although this work focuses on only two time points, D4 and D20 post-hatch, it represents part of a larger study where sampling of multiple time points was necessary.

### Analysis of phenotypic and i-STAT measurements

Phenotypic measurements and i-STAT blood values were analyzed using JMP Statistical Software (JMP®, Version 14.0.0). Pearson correlations were calculated between all measurements. Hierarchical clustering was performed and a heatmap generated for visualization. Given the small sample sizes of each day [7 and 10], a Shapiro-Wilk test was used to test each variable distribution for normality. To assess statistical significance of the differences between the two bird age groups, tests were done to compare measures of center. A 2-sample t-test was conducted on each variable meeting normality criteria, while a non-parametric 2-sample Wilcoxon test was performed for each variable that did not (PO_2_, BE, Na).

### Metabolome

Frozen liver plasma was shipped on dry ice to the UC Davis West Coast Metabolomics Center (Davis, CA) for untargeted metabolomics analysis. Primary metabolism analysis was done with gas chromatography-time of flight/mass spectrum (GC-TOF MS) and reported as peak heights normalized to mean total ion current (mTIC), or average total metabolome levels for each tissue. A total of 657 compounds were reported. Two hundred five were identified by name, while 452 were assigned BinBase database identifiers. Analysis of metabolome data was done using Metaboanalyst [58–60]. Statistical analysis was performed on all samples (11 D4, 12 D20). Variables with very small values were considered to be non-informative and were filtered using median intensity value. Remaining data was log transformed and Pareto scaled. Principal component analysis (PCA) and random forest were performed. Compounds that differed in level between bird age groups were identified by fold change and t-test using a log_2_ fold-change cutoff of 1, equal variances, and an adjusted *p*-value cutoff of 0.1. Pathway enrichment analysis was done for each day separately using curated compound names. Compounds used were determined by t-test (*p* < 0.05), resulting in 37 at D4 and 41 at D20. Fisher’s Exact Test was used for overrepresentation, out-degree centrality, the *Gallus gallus* pathway library, and the default background. For KEGG Search and Color Pathways search [23], InChiKeys provided by UC Davis for each metabolite were matched with compounds with the same isotopes but varying stereochemistry using PubChem Identifier Exchange [61] to obtain KEGG Compound IDs. Thus, compounds would have the same mass to charge ratio but vary in structure, allowing for chemically similar forms of metabolite compounds (i.e. different conformations of sugars).

### Transcriptome

Total and small RNA was isolated from each frozen liver sample using mirVana miRNA Isolation Kit with phenol (Thermo Fisher Scientific, AM1560) and DNAse treated using DNA-*free*™ DNA Removal Kit (Thermo Fisher Scientific, AM1906) according to the manufacturer’s protocols. After each step, RNA concentration and purity was tested using a NanoDrop spectrophotometer (NanoDropTM 1000, Thermo Scientific). Sample quality was further assessed at the University of Delaware Sequencing & Genotyping Center (Delaware Biotechnology Institute, Newark, DE) using an AATI Fragment Analyzer. Samples meeting a threshold of RNA Integrity Number (RIN) > = 7.0 were retained for library preparation. In total, 10 samples from D4 and 7 samples from D20 met the cutoff. cDNA libraries were prepared and indexed using Illumina TruSeq Stranded mRNA Library Prep Kit (Illumina, RS-122-2101) according to the manufacturer’s protocol. Final library concentration was assessed using Qubit dsDNA BR Assay Kit (Thermo Fisher Scientific, Q32850) and Qubit fluorometer 2.0 (Thermo Fisher Scientific). Libraries were pooled in groups of eight and paired-end sequenced at DBI on an Illumina HiSeq 2500 using one lane per pool and generating read lengths of 51 base pairs (bp).

Fastq files were down-sampled to 15,000 reads each using seqtk [62] to dilute any batch effect. Read quality was analyzed both before and after trimming using FastQC v0.11.7 [63]. Trimming was done using TrimGalore v0.4.5/CutAdapt v1.16 [64, 65]. Paired-end reads were mapped to Ensembl galGal6 (GRCg6a) [66] using splice-aware mapping software HiSat2 v2.1.0 [67] and converted to sorted bam files with SAMtools [68]. Transcripts and gene abundance in both fragments per kilobase of transcript million mapped reads (FPKM) and transcripts per million (TPM) were quantified with Stringtie v1.3.4d [69–71] using Ensembl reference annotation version 95 [66]. Raw counts were quantified with featureCounts v1.6.0 [72].

Differential expression was performed using statistical software R DeSeq2 package [73, 74]. DeSeq2 uses Benjamini-Hochberg (FDR) correction for multiple hypothesis testing to adjust *p*-values [75]. Genes meeting significance threshold of adjusted p-value < 0.05 from each method were compared for agreement, yielding 945 genes more highly expressed in D4 and 1265 genes more highly expressed in D20.

### Ontology & pathway enrichment

Differentially expressed genes (DEG) were analyzed for gene ontology and pathway enrichment analysis using DAVID 6.8 [20] and PANTHER [76, 77]. Significant DEG (adjusted *p* < 0.05) for each day were converted to NCBI gene IDs using bioDBnet [78] and put into DAVID for ontology analysis. One thousand three hundred forty-two gene IDs were recognized for D4, and 1703 for D20. Default parameters were used, except for gene ontology categories, “FAT” was chosen rather than “Direct.” Ontology was also analyzed using official gene symbols to take advantage of the improved annotation quality for human genes. One thousand two hundred eighty-eight gene IDs were recognized for D4 by this method, and 1495 for D20. Differentially enriched pathways were identified using KEGG [23], Reactome [79], and STRING [22].

### Principal component analysis

Stringtie outputs gene abundance in transcripts per million (TPM). Genes were filtered, retaining those with values of at least 0.1 in 7 or more samples. Principal component analysis (PCA) was done using these 13,117 genes and R packages FactoMineR [80] and factoextra [81].

### Metabolic pathway analysis

Differentially expressed genes and differentially abundant metabolites were used in KEGG Search & Color pathway tool to identify specific areas of pathways that may be upregulated in each day. NCBI gene IDs from all significant DEG (adjusted *p* < 0.05, total 3065 genes) and KEGG compound IDs from metabolite analysis (243 compounds) were used to search against *Gallus gallus* pathways in KEGG Search and Color Pathways.

## Supplementary Information

**Additional file 1.**

**Additional file 2.**

**Additional file 3.**

**Additional file 4.**

**Additional file 5.**

## Data Availability

The transcriptome dataset supporting the conclusions of this article is available in the FAANG and EMBL-EBI European Nucleotide Archive, [Study Accession: ERP128609, https://www.ebi.ac.uk/ena/browser/view/PRJEB44547]. The processed transcriptome data (TPM) and raw metabolome data is also included within the article and its additional files (Table S3 - transcriptome (TPM), Table S4 - metabolome (peak intensities).
